# A Possible Mechanism for Evading Temperature Quantum Decoherence in Living Matter by Feshbach Resonance

**DOI:** 10.3390/ijms10052084

**Published:** 2009-05-13

**Authors:** Nicola Poccia, Alessandro Ricci, Davide Innocenti, Antonio Bianconi

**Affiliations:** Department of Physics, Sapienza University of Rome, P. Aldo Moro 2, 00185 Roma, Italy; E-Mails: nicola.poccia@roma1.infn.it (N.P.); alessandro.ricci@uniroma1.it (A.R.); innocenti1@libero.it (D.I.)

**Keywords:** origin of life, quantum coherence, biological networks, network of networks, Bose condensation, quantum statistics of networks, Feshbach resonance, multiband superconductivity, multigap superconductivity

## Abstract

A new possible scenario for the origin of the molecular collective behaviour associated with the emergence of living matter is presented. We propose that the transition from a non-living to a living cell could be mapped to a quantum transition to a coherent entanglement of condensates, like in a multigap BCS superconductor. Here the decoherence-evading qualities at high temperature are based on the Feshbach resonance that has been recently proposed as the driving mechanism for high T_c_ superconductors. Finally we discuss how the proximity to a particular critical point is relevant to the emergence of coherence in the living cell.

## 1. Introduction

In these last years the genomes of many species have been sequenced, the structures of many macromolecular machineries of the cell have been solved using advanced physical methods and a large amount of experimental information has been accumulated on the networks of interactions between biomolecules in the living cell [1]. A consensus is growing that the emergence of the living cell from prebiotic phases of the matter is related with the onset of a particular macroscopic coherent biochemical reaction phase and the interaction with the surroundings leads to Darwinian evolution. In a recent review Ho [2] has presented important arguments for coherence in the cell. The works of Frölich [3] and Del Giudice *et al*. [4] stood at the origin of the physical search of coherence in living organism. The latter focussed on collective excitations in a biological system such as solitons and coherent electric waves and proposed that the living state is a practical realization of a Bose-condensate. Jaeken [5] connected these physical approaches to more physiological approaches towards coherence, such as those of Ling [6] and Pollack [7].

The challenge of the post-genomic era is to study how biological networks actually work in the space-time in living cells. This is described today as a coherent dynamical network of networks in a physical state where evolution has operated in lowering the network entropy [8,9].

The idea that the coherent state of living matter emerges in the proximity of a critical point in the so called scenario of biological order at the edge of chaos is attracting high interest in the scientific community. Mathematical models of evolving systems in which the rate of evolution is maximized near the edge of chaos have been proposed by Kauffman and Kadanoff [10]. In fact, the coherent phase of the living matter is where the complexity is maximal, between dynamical order and randomness or chaos. It has been proposed that the biological networks operate in a region where by changing a physical variable, as for example the proton concentration, they go through a behavior phase transition [11]. It has been shown that biological networks can be described by quantum statistics [12–14]. There is growing experimental evidence that quantum coherence could play a relevant role in the cell [15,16], and it is possible that the dynamic ordered state in the living cell is a manifestation of a quantum coherence [17,18].

The superfluid phases, the Bardeen Cooper Schrieffer (BCS) condensate for a fermionic gas and the Bose-Einstein condensate for a bosonic gas are clear realizations of macroscopic quantum coherence [19]. In this scenario the key physical problem is how is it possible that the quantum coherence phase could resist the de-coherence attacks of temperature.

We propose to examine the problem of the emergence of biological order as a form of quantum-like condensation in the way we progressively understand more from quantum matter studies in high T_c_ superconductors. We start from a picture of the minimum cell as a system made up of networks of biochemical reactions [20]. We are focusing our attention on the phase transition from non living to living matter for a system of biomolecules embedded in the architecture of a minimum cell. These types of phase transitions from a non living cell to a living one can occur, as a first example, by changing the temperature from below to above a critical temperature or as a second example, by changing the proton concentration above a critical value [11].

Clearly the living cell is a selected particular state of matter [21]. This particular state of matter has appeared in our Universe at a particular time and temperature. Therefore in this work we first discuss the emergence of life in the actual time scale of the Universe that only recently has been established. In the plot of the temperature versus time, elapsing from the Big Bang, we discuss the BCS-like phase transition giving the spontaneous symmetry breaking transition at the universe temperature of 10^12^ K and at 10^−4^ seconds after the Big Bang, then we discuss the earliest time possible for the transition from the non-living to living matter i.e., the emergence of life.

The living state of matter is associated with the first formation of nano-architectures in the cells made up of superlattices of quasi-2D water layers intercalated between lipid bilayers. This cell architecture reduces the dimensionality of the available space for the dynamical biochemical reactions embedded in the 1D superlattice to a quasi-2D space.

We propose that the quantum like transition that realizes the stable state of living matter at room temperature is similar to the *non conventional BCS-like transition* as seen in high T_c_ superconductors. In this quantum mechanism the many body Feshbach resonance for molecular association and dissociation processes is proposed to be effective for giving a macroscopic quantum coherent phase that avoids the temperature quantum de-coherence effects. This resonance requires nanoscale architectures in order to realize quantum condensates that are able to avoid temperature de-coherence effects [22,23]. In analogy with high T_c_ superconductors, we propose that the proximity to a particular quantum critical point [24–26] is related with the key role of the Feshbach resonances.

## 2. Mass Formation by a BCS-like Phase Transition at High Temperature in the Early Universe

A key piece of experimental information for understanding the emergence of living matter is to locate life in the time scale of the Universe. The quantitative measure of the time elapsed from the Big Bang to our days has been object of scientific discussion for a long time. Recently the age of the Universe has been carefully measured to be 13.69 ± 0.13·10^9^ yr by cosmic microwave background (CMB) data [27] and using Cepheids as the fundamental principal of extragalactic distances [28].

**Figure 1.**
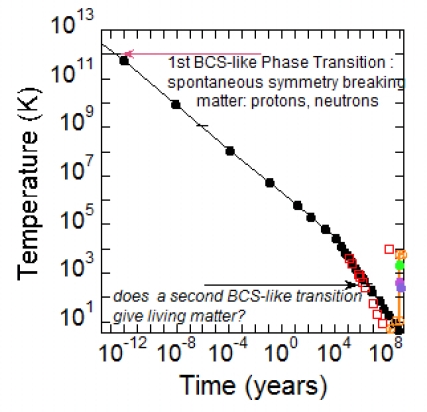
The temperature evolution of the Universe as a function of time in the early stage of the Universe after the Big Bang. The first BCS like phase transition occurring 10^−12^ yr after the Big Bang was the origin of nuclear matter as we know today. In this work we propose that by decreasing temperature around the boiling point of water, it is possible that a second BCS like phase transition occurred that was the cause of the origin of living matter as we know today.

The cosmic microwave background radiation measured by the COBE satellite provides a further indication about the temperature of the Universe nowadays [29]. The data of the thermal history of the Universe are reported in [30,31]. In Figure 1 we have plotted the temperature as a function of time on a logarithmic scale in order to show the temperature behaviour in the early stage of the Universe after the Big Bang.

It is thought that as matter cooled it underwent phase transitions, during the course of which the Universe's state of symmetry was altered. As the temperature of the Universe was decreasing to a temperature of 10^12^ K (at ~10^−4^ seconds after the Big Bang) the thermal energy of the universe was low enough to allow the hot soup of quarks to condense; quarks fell together in triplets under the influence of the strong nuclear force and have remained stable ever since, as protons and neutrons. This period is known as the quark-hadron transition.

Phase transitions from a disordered state into an ordered state with broken symmetry can occur in the real space (liquid-solid transition) or in the momentum space (BCS, Bose Einstein condensation). Kirznits and Linde [32] have proposed a phase transition theory where the masses of vector bosons and fermions are absent in the initial Lagrangian, and are generated by spontaneous breakdown of symmetry. This model forms an integral part of a unified theory of electromagnetic and weak interactions. In the standard picture of cosmology this phase transition is associated with a chiral symmetry breaking after the electroweak transition to convert a plasma of free quarks and gluons into hadrons.

The spontaneous symmetry breaking theory has evolved from the BCS theory, describing the formation of a quantum condensate of a fermionic gas being a phase transition from a disordered state of matter to a dynamically ordered state of matter. The BCS theory of superconductivity is a case of broken gauge invariance and therefore is a case of dynamical symmetry breaking [33]. In 1961 Nambu and Jona-Lasinio proposed a theory of elementary particles based on an analogy with the BCS theory of superconductivity [34] that was recognized by the scientific community with the 2008 Nobel Prize for Physics. They showed how fermion masses would be generated in a way that is analogous to the formation of the energy gap in a superconductor. This is where the idea of a spontaneous broken symmetry being the way in which the mass of particles could be generated first arose providing the physical base for the quark-hadron transition. The idea started from the striking analogy of the quasi-particles in superconducting condensates and the Dirac equation. In fact in the Weyl representation, the Dirac equations reads as in (1):

(1) $$\begin{array}{l} E\;\Psi_{1}=\vec{\sigma }\cdot \vec{p}\Psi_{1}+m\Psi_{2}\\ E\;\Psi_{2}=-\vec{\sigma }\cdot \vec{p}\Psi_{2}+m\Psi_{2} \end{array}$$

where Ψ_1_ *and* Ψ_2_ are the eigenstates of the chirality operator *γ*_5_, with eigenvalues given by (2):

(2) $$E=\pm \sqrt{p^{2}+m^{2}}$$

This result actually gives the dispersion of massive relativistic particles. In the BCS theory the electrons near the Fermi surface are described by equation (3):

(3) $$\begin{array}{l} E\;\Psi_{p,+}=\varepsilon_{p}\Psi_{p,+}+\varphi \;\Psi_{-p,-}^{+}\\ E\;\Psi_{-p,-}^{+}=-\varepsilon_{p}\Psi_{-p,-}^{+}+\varphi \;\Psi_{p,+} \end{array}$$

where Ψ *_p_*_,+_ *and* $\Psi_{-p,-}^{+}$ are the wavefunctions for an electron and a hole of momentum p and spin +, that has eingenvalues given by (4):

(4) $$E=\pm \sqrt{\varepsilon_{p}^{2}+\varphi^{2}}$$

This gives also the dispersion of Bogolyubov quasi-particles [35] with kinetic energy *ɛ_p_* in a superconductor with a superconducting gap Δ in the spectrum of a Fermi liquid.

The work of Nambu-Jona-Lasinio has produced a revolution in elementary-particle physics, since the protons and neutrons are view as composed by the quantum condensation of quarks (a particle with mass m is described as a BCS-like condensate with gap Δ) which give them peculiar properties.

The formal analogy between the masses of particles and the gaps of the BCS excitations spectrum provides a new point of view in seeing nucleons. The condensed matter physics of quarks is rapidly developing during these years by using methods of superconductivity theory adapted to Quantum Chromo Dynamics (QCD). This analysis results in the identification of condensates in diquark channels, analogous to the Cooper pairs of electrons in ordinary superconductor. This is known as the phenomenon of color superconductivity [36–38]. Because of the infinite degeneracy among pairs of quarks with equal and opposite momenta at the Fermi surface, an attractive interaction between quarks, even if arbitrarily weak, renders the Fermi surface unstable to the formation of a condensate of quark pairs. Creating a pair costs no free energy at the Fermi surface, while the attractive interaction results in a free energy benefit. The consequence of an attractive interaction between opposite momentum modes near the Fermi surface is therefore the formation of a condensate in the zero temperature ground state. We expect those quark quasiparticles which interact with the condensate to acquire an energy gap. The kink in the curve in Figure 1 about 400,000 years after the Big Bang when temperature dropped around 10^4^ K is associated with the formation of the first atoms.

## 3. The Emergence of Life in the Universe Timescale

A few million years after the start of the Universe, when temperature was about 10^3^K, the thermal energy of the Universe was below the dissociation energy of molecular hydrogen (13.6 eV), therefore only after this time could molecules be formed. The CMB temperature reached 370 K, the boiling point of water, at 3·10^7^ yr from the Big Bang. Therefore only after this time the formation of liquid water, considered to be the essential medium for biochemical interaction, becomes possible.

The living matter that we know in our planet is made of 23 different elements forming biomolecules in liquid water. Since the element synthesis occurs at the end of the life time of a star (as in a supernova explosion) the coexistence of the 23 elements in the same spot of the space becomes possible in the universe only at the end of the life of the first stars (about 1.5·10^9^ yr).

The Earth, where all 92 stable elements are present, is a product of a supernova explosion that was trapped by the sun, at about 9·10^9^ yr after the Big Bang [39–41]. It was a molten ball at 2,000 K that was periodically vaporized and covered with a 2,000 K rock vapour atmosphere when meteoritic activity decreased.

**Figure 2.**
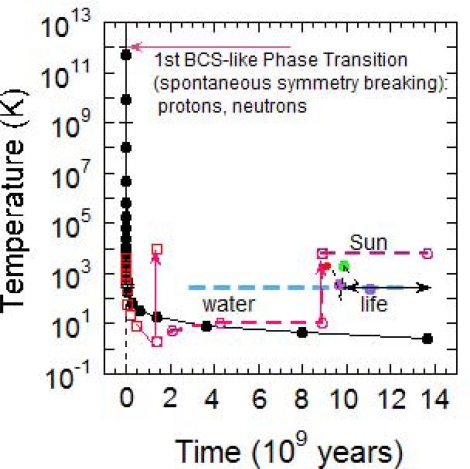
The CMB temperature (solid line and filled dots) as a function of time in linear scale in the Universe and the hydrogen gas temperature (squares) with the discontinuities (arrows) for the formation of first stars and the sun. The earth formation and the onset of life dots with vaporization periods of the Earth surface by meteorites. Green and blue filled dots indicate the appearance on Earth of thermophile and cyanobacteria respectively.

It is possible that forms of primitive life, like thermophile bacteria, which can survive and reproduce until 394 K [42], appeared on the Earth surface 9.6·10^9^ yr after Big Bang. Late accretion impacts may have killed off life on our planet as late as 9.8·10^9^ yr after Big Bang [43]. At present, we can say that life had evolved as early as 10.1·10^9^ yr after Big Bang, taking into consideration the available data, as shown in Figure 2 [44–46].

Due to this uncertainty, we suggest that non-living to living matter transitions happened in the range of time when on Earth the water was in the range of temperature of 320 K and the Universe age was 9.8 ± 0.43·10^9^ yr. However, when temperature decreased new forms of life found evolutionary advantage over the others until the complex cyanobacteria were formed around 11 ± 0.4·10^9^ yr after the Big Bang [47]. If life emerges as a consequence of a phase transition, the same critical state could be reproduced several times on Earth and therefore it is possible that evolution have had multiple terrestrial genesis events [48,49].

## 4. An Early Ubiquitous Biological Nano-architecture: The 1D Superlattice of Membranes

**Figure 3.**
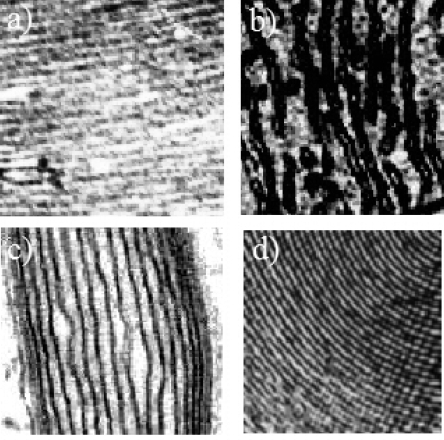
The superlattice of water layers intercalated by lipid bilayers measured by SEM: a) in the chloroplast; b) in the mitochondrion; c) in myelin and d) in the Golgi apparatus.

According with Oparin the emergence of living matter is associated with the formation of the smectic 2D liquid of phospholipids forming the bilayer lipid membranes [50]. In living matter there are ubiquitous architectures made up of lipid membranes. In Figure 3 we show four common membrane architectures in biological matter showing the one dimensional (1D) order of quasi two dimensional (2D) layers. Panel a) shows the thykaloid membrane in chloroplast; Panel b) the inner and outer lipid membranes in the mitochondrion; Panel c) the myelin multilayered structure; Panel d) the inner multilayered structure of the Golgi. It is interesting seeing the similarity of the geometrical structure the lipid bilayers in spite of the different biological networks embedded in these nano-architectures performing quite different biological functions.

We propose that the role of the multilayer geometry is to lower the dimensionality for the dynamics of the biomolecules embedded in the system, giving rise to new form of interactions resulting in a quantum-like coherent behaviour which is the theme of the following chapter.

**Figure 4.**
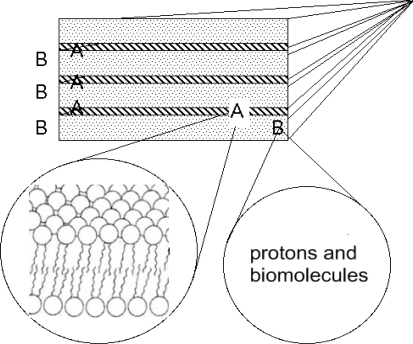
The schematic picture of the model made of a superlattice of lipid bilayers (portion A) intercalated by liquid water layers with itinerant biomolecules and protons (portion B).

These kinds of complex systems, having layered periodicities in the real space, give rise to different types of particles: a first one that moves only in the 2D space between the layers and a second one for which the hopping between layers is possible and therefore can have 3D character. In this scenario the evolution should have refined specific gates that allow the transfer of protons through the membrane. We can therefore schematize in Figure 4 the multilayered biological membranes as a superlattice of two types of layers B intercalated by layers A. The layers B are made of quasi-two dimensional water layers, having in solution protons and biomolecules. The layers A are made of hydrophobic lipid bilayers.

## 5. Biological Networks and Quantum Statistics

A living organism can be viewed as a collection of biochemical activities. Most biological characteristics arise from complex interactions between the numerous cell constituents, such as proteins, DNA, RNA and small molecules. Various types of interaction webs, or networks, (including protein-protein interaction, metabolic, signalling and transcription-regulatory networks) emerge from the sum of these interactions.

The emerging paradigm relates the biological functions with these biological networks [1]. This a novel description that could bridge the gap between genotype and phenotype. The cell is viewed as an overlay of a few biological networks made up of modules. Among them the most studied are the networks which involve protein-protein, protein-DNA and protein-metabolite interactions [51,52]. None of these networks are however independent, instead they form a *network of networks* that is responsible of the behaviour of the cell [1]. The smallest and the simplest living cell (called the minimum cell) is made of several functional networks which together forms a network of networks. The available Mycoplasm genome is therefore of relevance for the understanding of how a network of networks work to transform a non living thing in a living cell [53].

**Figure 5.**
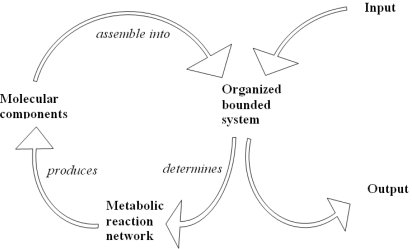
The cyclic process of metabolic biochemical reactions.

The metabolic network it is made up of a closed cycle of biochemical reactions as shown in Figure 5 by a graphic representation of a closed path and free metabolites. The free input metabolites interact with the closed path of reactions forming the network and are scattered out as free output metabolites [54].

It is clear the key problem for the emergence of life is the coherent dynamics of biological networks interacting with free biomolecules. The recent advances in this field have shown that biological complex networks can be described by quantum statistics [55–57] i.e., the Fermi distribution, or the Bose-Einstein distribution, or mixed Fermi and Bose statistics. The current focus in the network theory is therefore essentially on cooperative phenomenon and on dynamic processes taking place in networks [58–63]. In 2001 the Bose-Einstein-like condensation [12] was discovered in the fitness model of growing networks with nodes having different fitness for acquiring new links. In this phase transition for scale free networks few nodes of the networks grab a finite portion of all the links up to the point where under certain conditions, a single node grabs a finite fraction of all links, corresponding to Bose Einstein condensation in Bose gases. The dynamics of this off-equilibrium phase transition relevant for biological systems is the object of active ongoing research [14,59–61]. The condensation could appear also in the flux of elements such as metabolites in a metabolic network [62,63]. The networks describing simple ecosystems [61,64,65] can be described by Bose and Fermi-Dirac statistics and they may undergo a first or a second order phase transition akin to Bose-Einstein condensation for a non-interacting gas. Recently Garlaschelli *et al*. have described networks using generalized Bose and Fermi statistics [58] and have shown that they can undergo different kind of condensations towards ordered phases. The recent advances in the entropy of biological networks [8,9,66] will allow the measure of the emergence of biological order in the networks [67].

Let us consider a living cell constituted by many networks exploiting different functions which are not independent. The emergence of life in the cell is related to the cooperative dynamics of the network of networks [1], within a given network by the cooperative behaviour of its modules, and within the network modules the cooperative behaviour of biochemical reactions.

A possible scenario is that each of these networks is in the proximity (each one at a different distance) of off-equilibrium phase transitions toward ordered phases analogous to a quantum-like condensate and the life will emerge from the onset of coherence between condensates. In fact living matter displays a dynamical and spatial order with collective properties and non-local interactions like in a superfluid [67–69]. One of the problems in this scenario is that quantum coherence in known superfluids is destroyed by temperature de-coherence effects at room temperature. Therefore research has been focusing on the quantum tricks for the decoherence-evading qualities at room temperature. While most of the work has been done on mapping phase transitions in bosonic networks to the Bose-Einstein condensation (BEC) it is possible that fermionic networks describing evolving networks could undergo transitions that could be mapped to a BCS-like condensation. Therefore coherent phases mapped to BCS condensates could also be possible and help our understanding of the emergence of the Darwinian evolving life. In the following section we show that the new physics emerging in high T_c_ superconductivity are providing, by analogy, a new scenario for the origin of life showing a novel case of cross-fertilization between physics and biology.

## 6. The Multigap BCS Superconductivity

The BCS provides a general theory for condensation in fermionic systems [70–72]. There are many different types of BCS condensates which depend on the actual attractive interaction (Cooper pairing involving phonons is only one of them, among many possible cases), on the material architecture and on the position of the chemical potential. In this last twenty five years of research in high T_c_ superconductivity a plethora of BCS condensates have been proposed in order to search a particular one that allows the system to avoid temperature decoherence effects.

Recently looking for unity in the diversity of several recently discovered different types of high T_c_ superconductors: cuprates, [73] diborides [74] and iron pnictides, [75] appears that a particular BCS condensate is characteristic of high T_c_ superconductors: multigap superconductivity [22,23,76–80]. In these works it is shown that in the multiband or multigap theory of superconductivity the quantum ordered phase is made of many condensates with different order parameters Δ*_i_*. In this emerging new paradigm the key term is the interband coupling between condensates that is an exchange like interaction driven by the coulomb field without a mediator. In fact this is an interference effect among the condensates which can be negative or positive.

It is well known that the critical temperature of a superconducting phase is lower than 20K in a homogeneous three-dimensional system [76–78], but now the high T_c_ superconductors show a common heterostructure made of a superlattice of layers. Therefore the research is now shifting toward the identification of a particular nanoscale heterogeneity suppressing decoherence effects at high temperature.

The key for solving the problem has been to focus on subtle structural and dynamical details that could give a macroscopic quantum coherence that is strong enough to be stable at room temperature. Therefore one has to go beyond the standard BCS approximations for a generic homogeneous system: (i) *the high Fermi energy*: where the Fermi energy is assumed at an infinite distance from the top or the bottom of the conduction band, (ii) *the isotropic approximation*: the pairing mechanism is not electronic state dependent. The BCS wave-function (5) of the superconducting ground state has been constructed by configuration interaction of all electron pairs (+k with spin up, and −k with spin down) on the Fermi surface in an energy window that is the energy cut off of the interaction:

(5) $$|\Psi_{BCS}\rangle =\prod_{k}\left(u_{k}+v_{k}c_{k↑}^{+}c_{-k↓}^{+}\right)|0\rangle$$

where |0〉 is the vacuum state, and $c_{k↑}^{+}$ is the creation operator for an electron with momentum k and spin up. The Schrieffer idea of this state with off-diagonal long range order came from the configuration interaction theory by Tomonaga involving a pion condensate around the nucleus [81].

In *anisotropic multigap superconductivity* one has to consider configuration interaction between pairs, in an energy window ΔE around the Fermi level, in different locations of the k-space with a different pairing strength, that gives a k-space dependent superfluid order parameter i.e., a k-dependent superconducting gap. A particular case of anisotropic superconductivity is multigap superconductivity, where the order parameter and the excitation gap are mainly different in different bands.

The theory of multiband superconductivity, including the configuration interaction of pairs of opposite spin and momentum in the *a_i_*-bands was developed by Kondo [82] on the basis of the Bogolyubov transformations [35,83] where the many body wave function is given by (6):

(6) $$|\psi_{Kondo}\rangle =\prod_{i}\left[\prod_{k}\left(u_{i,k}+v_{i,k}a_{i,k↑}^{+}a_{i,-k↓}^{+}\right)\right]|0\rangle$$

The element corresponding to the transfer of a pair from the *a_i_*-band to the *a_j_*-band or vice versa appears with the negative sign in the expression of the energy. This gain of energy is the origin of the increase of the transition temperature driven by interband pairing.

The “interchannel pairing” or “interband pairing” that transfers a pair from the “a_i_”-band to the “a_j_”-band and *vice versa* in the multiband superconductivity theory is expressed by the off diagonal element:

(7) $$\sum_{k,k\prime }J(k,k^{\prime })(a_{i,k↑}^{+}a_{i,-k↓}^{+}a_{j,-k↓}a_{j,k↑})$$

where a_i_^+^ and a_j_^+^ are creation operators of electrons in the “a_i_” and “a_j_” band respectively and a_i_ and a_j_ are destruction operators of electrons in the “a_i_” and “a_j_” band respectively and *J*(*k*,*k*′) is an exchange-like integral. This interband pairing interaction may be repulsive as it was first noticed by Kondo. Therefore it is a non-BCS pairing process since in the BCS theory an attractive interaction is required for the formation of Cooper pairs. Another characteristic feature of multiband superconductivity is that the order parameter shows the sign reversal in the case of a repulsive interband pairing interaction.

The advances in this field are related with the development of the theory of configuration interaction between different excitation channels in nuclear physics including quantum superposition of states corresponding to *different spatial locations*. The resonances in nuclear scattering cross-section [84,85] for the processes of neutron capture and nuclear fission in the cloudy crystal ball model of nuclear reactions have been called Feshbach resonances. These resonances in the scattering deal with configuration interactions in multi-channel processes involving high energy bound states degenerate with the continuum. These are a generalization of the “shape resonances” related with the configuration interaction theory for autoionization processes in atomic physics [86] and Majorana theory of quasi-stationary states [87]. These resonances between the discrete and continuum energy spectrum can be carefully tuned by engineering the architecture.

These resonances are a clear manifestation of the non locality of quantum mechanics and appear in many fields of physics and chemistry such as the molecular association and dissociation processes. In the physics of ultra cold gases the Feshbach resonances show up by tuning the chemical potential of the atomic gas around the energy of a discrete level of a diatomic molecule controlled by an external magnetic field. The Feshbach resonances is a quantum phenomenon that enhances the entanglement of the particles in the continuum therefore they have been used to achieve the Bose-Einstein condensation in the dilute bosonic gases of alkali atoms and BCS-like condensate in fermionic ultra-cold gases with large values of T_c_/T_F_.[88,89].

The process for increasing T_c_ by a Feshbach “shape resonance” in a superconductor was first proposed for a superconducting thin film by Blatt and Thompson [90–92] in 1963. The resonance described by Blatt occurs in a superconducting thin film of thickness L where the chemical potential crosses the bottom E_n_ of the n-th subband of the film, a quantum well, characterized by *k_z_* = *nπ/L* with n>1.

Following the experimental evidence of nanoscale striped lattice fluctuations in cuprates, [93] and the proximity to a structural phase transition to a charge ordered phase that competes with superconductivity [94–97] it has been proposed to increase T_c_ via a Feshbach resonance in heterostructures made of superlattices [22,23,98,99].

**Figure 6.**
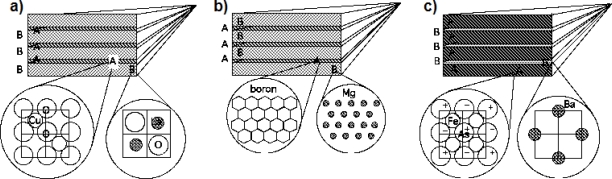
Multilayered architectures formed by a superlattice of metallic superconducting units intercalated by a different material in three different high T_c_ superconductor families: a) cuprates, b) diborides, c) pnictides.

The discovery of diborides [74] in 2001 and of the FeAs based high T_c_ superconducting multilayers [75] in 2008, has shown that they have a similar nanoscale architecture as that of cuprates, as shown in Figure 6. These results have pointed toward the relevance of a multilayer structure for the emerging quantum macroscopic coherence.

The idea is that the particular multilayered architectures formed by a superlattice of metallic superconducting units intercalated by a different material can bestow decoherence evading qualities to the system. The quantum tricks to realize high T_c_ superconductors are based on the generic feature of the electronic structure of the superlattices.

In the superlattice the electronic structure is made of subbands where n and n′ are the subband indexes. k_x,y_ (k_x’,y′_) is the component of the wavevector in the plane direction (or longitudinal direction) and k_z_, (k_z′_) is the superlattice wavevector (in the transverse direction) of the initial (final) state in the pairing process, and μ the chemical potential.

In the BCS approximation, i.e., a separable kernel, the gap parameters have the same energy cut off as the interaction. The value of the gaps Δ*_n_* (*k_z_*) around the Fermi surface in a range ħ*ω*_0_ depend on the subband index and the superlattice wavevector *k_z_*. The ground state energy gap in each subband Δ*_n_* (*k_z_*) depends form the gaps in other subbands according with the self consistent equation (8) [99,100]:

(8) $$\Delta_{n}(\mu ,k_{z})=-\frac{1}{2N}\sum_{n^{\prime }k_{z}^{\prime }k_{x,y}^{\prime }}\frac{V_{n,n^{\prime }}(k,k^{\prime })\;\Delta_{n^{\prime }}(k_{z}^{\prime })}{\sqrt{{\left(E_{n^{\prime }}(k_{z}^{\prime })+\varepsilon_{k_{x,y}^{\prime }}-\mu \right)}^{2}+\Delta_{n^{\prime }}^{2}(k_{z}^{\prime })}}$$

where N is the total number of wavevectors. This equation solved iteratively provides a set of anisotropic gaps strongly dependent on the subband index and weakly dependent on the superlattice wavevector k_z_. The superconducting gap of the n band shows a shape resonance when the chemical potential is tuned near the bottom of the n+1 subband and vice versa. The width of the resonance is controlled by the interaction cut off and the superlattice dispersion in the z direction. The critical temperature T_c_ of the superconducting transition is obtained by the linearized BCS equation (9):

(9) $$\Delta_{\text{n}}(\text{k})=-\frac{1}{\text{N}}\sum_{{\text{n}}^{\prime }{\text{k}}^{\prime }}{\text{V}}_{\text{n}{\text{n}}^{\prime }}(\text{k},{\text{k}}^{\prime })\frac{\text{tgh}(\frac{\xi_{{\text{n}}^{\prime }}({\text{k}}^{\prime })}{{\text{2T}}_{\text{c}}})}{2\xi_{{\text{n}}^{\prime }}({\text{k}}^{\prime })}\Delta_{{\text{n}}^{\prime }}({\text{k}}^{\prime })$$

where ζ_n_(k)=ɛ_n_(k)-ɛ_F_.

In the multigap superconductivity for a two band system the coupling interaction is given by a matrix (10) [80]:

(10) $$\lambda =\left|\begin{array}{cc} \lambda_{1,1} & \lambda_{1,2}\\ \lambda_{2,1} & \lambda_{2.1} \end{array}\right|$$

where *λ*_1,1_ *and λ*_2,2_ are the intraband pairing terms that are at the origin of single BCS condensates in the electronic system (1) and system (2) respectively when they are well separated. The non diagonal terms *λ*_1,2_ *and λ*_2,1_ are the interband pairing terms that control the entanglement between the two condensates. It is not necessary that both systems have a non zero coupling term for multiband superconductivity in fact it is possible that only one isolated system forms a condensate since the condensation can be induced in this system by the interband non diagonal terms.

A simple formula of the critical temperature T_c_ where the Fermi level is far apart from the band edges for a two band system with density of states *N*_1_ *and N*_2_, intraband couplings *λ*_1,1_ *and λ*_2,2_ and interband coupling *λ*_1,2_ is given by (11):

(11) $$\frac{k_{B}T_{c}}{\hbar \omega_{0}}=1.14\;\text{exp}\left\lfloor -\frac{A-B}{\lambda_{1,2}^{2}-\lambda_{1,1}\lambda_{2,2}}\right\rfloor$$

where:

$$A={\left[\frac{\lambda_{1,2}^{2}}{N_{1}N_{2}}+\frac{1}{4}{\left(\frac{\lambda_{2,2}}{N_{1}}-\frac{\lambda_{1,1}}{N_{2}}\right)}^{2}\right]}^{1/2}$$

and:

$$B=\frac{1}{2}\left(\frac{\lambda_{2,2}}{N_{1}}+\frac{\lambda_{1,1}}{N_{2}}\right).$$

It is clear from this expression that where A=B there is divergent enhancement of the critical temperature that will reach the energy scale of the interaction cut off and therefore it could easily reach 400K.

In this scenario the Feshbach resonance [100,101,22] occurs in the quantum interference between the pairing channels and is related with the matrix element for the interband pairing term. It occurs where the chemical potential is increased to a point where the additional particles are injected in a second new system. Therefore the Feshbach resonance occurs at quantum critical point where the particles start to occupy a new system. In the field of growing networks it will correspond to the point where the additional nodes added to the system start to populate a second small strongly interacting network. The relevant aspect of the Feshbach resonance is that the system at the resonance forms a stable resistant multigap condensate, where the quantum interference is positive and its critical temperature is very high, but the system is close to an instability where the quantum interference is negative and the critical temperature is very low.

This phase of matter is expected near a quantum critical point. This scenario is supported by the recent remark that in the biological matter embedded in membrane stacks the long range Coulomb interaction drives the mobile charges close to the Wigner crystal phase [102] like in high T_c_ superconductors with the formation of polaron strings [103]. In these conditions the joint elastic and coulomb interactions drives the system to a multiscale phase separation [104,25].

## 7. Feshbach Resonances and Quantum Coherence in Living Matter

The origin of life is related with the onset of a coherent phase of a finite number of selected biomolecules making the living cell. The cell is made of few essential biological networks represented as graphs where nodes are connected by links to their experimentally determined partners [1]. In the protein-protein interaction networks the nodes are proteins and the links are biochemical reactions. Here we propose that the biochemical reactions selected by evolution are controlled by Feshbach resonances. These quantum resonances have been recently observed in gas phase chemical reactions. This important advance in the field of chemical reaction [105–112] dynamics has identified resonances, that are transient intermediate states produced during the reaction, occurring near the top of a reaction barrier (the “transition state”), where one would not normally expect to find metastable states. These new studies provide unequivocal evidence for these non-intuitive resonance states and shed light on how molecular quantum states influence reactivity in chemical reactions. A substantial progress has been made in developing theory to account for quantum effects on chemical reactions [105–109]. Crossed molecular beam and laser experiments are enabling reactive transitions to be measured to an increasing precision and Feshbach and shape resonances have been detected in F+HD->HF+D and F + H_2_-> HF + H [110–112]. The individual quantum states of the reactant molecules influence the rate of reaction and which products are produced.

These results have produced relevant advances in understanding enzyme kinetics that have allowed for a detailed understanding of how many enzymes work. It is now appreciated that enzyme-catalyzed proton and hydride transfer reactions often occur by quantum mechanical tunneling [113–115]. For example hydrogen transfer reactions catalyzed by coenzyme B12 dependent methylmalonyl-CoA mutase shows very large kinetic isotope effects [115], indicating that they proceed by a highly quantum tunneling mechanism.

The Feshbach resonances have been discovered in the early period of nuclear physics and used to study the nuclear levels spectrum [87]. The energy spectrum of particles emitted in a nuclear reaction has two quite distinct regions, namely the low energy discrete region in which well-separated bound levels may be distinguished and the higher energy continuum region in which the levels are unbound or virtual and overlap considerably. The discrete structure of the spectrum survives for the lowest virtual levels and may be seen as individual resonances (shape resonances) in appropriate reactions. These very narrow resonances are the result of the formation of a long-lived compound nucleus at high energy during the scattering process, with a binding energy close to that of the incoming particle.

A Feshbach resonance for biomolecules is an enhancement in the scattering amplitude of a first biomolecule incident on a second biomolecule when the energy of the former is approximately that needed to create a quasibound state of the two-particle system. If a pair of biomolecules has a molecular bound state near zero energy, then during collisions they stick together for a little while as they undergo a Feshbach resonance. In a few words, a Feshbach-resonant molecular collision occurs when two biomolecules collide and form a long-lived transient molecule, which ultimately decays again in two free biomolecules. In the living cell the long-lived transient protein-protein quasi-bound states should be predominantly determined by the spectrum of protein conformation fluctuations.

The essential biological networks making up a living cell include: protein biosynthesis, ribosome biogenesis, metabolism, transport, cell homeostasis, cell cycle. In the protein-protein interaction network the nodes are proteins and the links are biochemical reactions [116] while in the metabolic network nodes are small molecules (substrates) and links are the chemical reactions controlled by specific enzymes [117]. These recent studies reveal that protein-protein interaction networks are not randomly organized and display properties that are typical of hierarchical networks, combining modularity and local clustering to scale free topology. The open problem is the dynamic nature of protein interactions inside the cell. In the protein-protein network two proteins may interact and form a new complex depending on whether they are linked by a link. Links in networks therefore suggest a transient presence of an associated state between two proteins. If a node A has more than a link, for example two links, this means that the same protein (node A) can associate and dissociate at subsequent times with two different proteins B and C. Each link is weighted by the proper equilibrium reaction constant which describes quantitatively the rate of association and dissociation:

A+B+C→B+AC*→BA*+C→A+B+C

In this simple example therefore we have two different quasi-bound states AC* and BA* for the same protein A and each of these states is characterized by an association-dissociation equilibrium constant.

The links attached to the node can belong to the same network or be in a modular region between overlapping networks each of them performing a variety of tasks. However, when a first node protein is transiently associated with a second linked protein, the third protein remains dissociated and free in solution. But how can we try to explain the dynamical process which forbids for the free proteins to bind with the many other highly concentrated proteins in a typical cell milieu? We propose that the protein-protein interaction has to be tuned (changing physical parameters such as density, temperature, membrane architectures, pH and temperature) near resonating Feshbach states. In this resonating state it is of not main importance how much far away the protein is when it is dissociated because it will return to associate transiently again. The coherence in the dynamics of proteins inside the cell is related with the Feshbach resonances of the biological reactions so that the network develop if the same protein associate and dissociate transiently in time with a great number of proteins. In other word, this means that the protein is an highly linked node of the network. The link condensation on a node protein can be controlled weighting the link themselves with appropriate association and dissociation constants. A carefully engineering of the dissociation constants within a network of protein interactions could be a way to achieve a Feshbach resonance in biological matter, establishing finally a robust and functional system against the decoherence attacks of temperature. The interesting physics is that the living cells will be a condensate of condensates as in high T_c_ superconductors.

## 8. Conclusions

In this work we have located the origin life on the timescale of our Universe. We have presented a scenario where the emergence of life is associated with an entangled phase of networks. Therefore it is possible that in the evolution the coherent states of biological matter, appeared as coherent phase of matter, could be mapped as multigap BCS condensates.

Until very recently the coherence of living organisms, despite its very central importance for life, was an almost totally neglected property of living organisms by the biochemical oriented scientific community. Right now this situation starts to change focusing on how the coherence of living matter got established at the onset of life. This review proposes a mechanism based on the knowledge of new achievements in physics. The origin of this coherence is connected to a phase transition at a critical point, where the system is initiated and after which the system has sufficient stability to continue its existence. The fundamental question is posed how the system could escape the decohering activity of high temperature (the real problem to overcome) and the new physics emerging in high T_c_ superconductors is used as a reference physical system.

Quantum coherence relevant to biology always appears in the normal ambient at the normal temperatures in conjunction with a well defined energy flow. The scenario presented here show that the dynamical biological order in the living cell requires the presence of constant energy flow in fact the Feshbach resonance is related with a high energy quasi bound state degenerate with an high energy continuum of free particles. A lot of open problems have to be faced to answer the Erwin Schrödinger question: “What is life?” [118] at the end of the first decade of the XXI century but now the crucial issue is well defined and the development of interdisciplinary research, getting the benefit of cross-fertilization between different fields, is driving us to unexpected surprises.
